# IFN-γ transforms the transcriptomic landscape and triggers myeloid cell hyperresponsiveness to cause lethal lung injury

**DOI:** 10.3389/fimmu.2022.1011132

**Published:** 2022-09-20

**Authors:** Atul K. Verma, Michael McKelvey, Md Bashir Uddin, Sunil Palani, Meng Niu, Christopher Bauer, Shengjun Shao, Keer Sun

**Affiliations:** ^1^ Department of Microbiology and Immunology, University of Texas Medical Branch, Galveston, TX, United States; ^2^ Department of Experimental Pathology, University of Texas Medical Branch, Galveston, TX, United States; ^3^ Department of Genetics, Cell Biology & Anatomy, University of Nebraska Medical Center, Omaha, NE, United States; ^4^ Department of Pathology and Microbiology, University of Nebraska Medical Center, Omaha, NE, United States

**Keywords:** Acute lung injury, cytokine storm, influenza A virus, methicillin-resistant *Staphylococcus aureus*, coinfection

## Abstract

Acute Respiratory Distress Syndrome (ARDS) is an inflammatory disease that is associated with high mortality but no specific treatment. Our understanding of initial events that trigger ARDS pathogenesis is limited. We have developed a mouse model of inflammatory lung injury by influenza and methicillin-resistant *Staphylococcus aureus* (MRSA) coinfection plus daily antibiotic therapy. Using this pneumonic ARDS model, here we show that IFN-γ receptor signaling drives inflammatory cytokine storm and lung tissue damage. By single-cell RNA sequencing (scRNA-seq) analysis, we demonstrate that IFN-γ signaling induces a transcriptional shift in airway immune cells, particularly by upregulating macrophage and monocyte expression of genes associated with inflammatory diseases. Further evidence from conditional knockout mouse models reveals that IFN-γ receptor signaling in myeloid cells, particularly CD11c^+^ mononuclear phagocytes, directly promotes TNF-α hyperproduction and inflammatory lung damage. Collectively, the findings from this study, ranging from cell-intrinsic gene expression to overall disease outcome, demonstrate that influenza-induced IFN-γ triggers myeloid cell hyperresponsiveness to MRSA, thereby leading to excessive inflammatory response and lethal lung damage during coinfection.

## Introduction

Acute respiratory distress syndrome (ARDS) is characterized by an overwhelming inflammatory response. The limited treatment option, with no effective therapy to prevent lung injury, has led to a mortality rate of approximately 40% in afflicted (1, 2). Hypercytokinemia, also known as “cytokine storm”, plays a central role in ARDS pathogenesis. As such, inflammatory cytokines TNF-α and IL-6 are significant predictors of severity and death of diseases including COVID-19 (3, 4). However, our understanding of initial events that trigger hypercytokinemia and ARDS is very limited.

Viral and bacterial pneumonia that activates excessive and dysregulated lung inflammation is the most frequent cause of acute lung injury (ALI) and ARDS (5, 6). Respiratory illness caused by influenza A virus (IAV) alone is generally self-limited. However, IAV infection predisposes hosts to secondary bacterial pneumonia, which is a major cause of hospitalization and death in influenza pandemics and epidemics (7–12). *Staphylococcus aureus* is one such opportunistic pathogen often implicated in post-influenza bacterial pneumonia. The increasing prevalence of methicillin-resistant *S. aureus* (MRSA) in communities and healthcare settings further complicates the problem. Particularly, IAV/MRSA coinfection can quickly develop into severe, necrotizing pneumonia with over 50% mortality, despite available flu vaccines, anti-viral drugs, and antibiotics (9, 12, 13).

Through post-influenza MRSA infection plus daily antibiotic treatment, we have developed a mouse model of ARDS that mimics the poor outcome of patients with influenza-complicated bacterial pneumonia. We show in this model that antibiotic therapy restores bacterial control but is not sufficient to rescue animals from inflammatory lung damage (14). Using this coinfection-induced ARDS model, we have shown that phagocyte oxidative burst promotes necrotic death of inflammatory cells, thereby resulting in lethal damage to surrounding tissue (15). Furthermore, we have demonstrated that the overproduction of proinflammatory cytokine TNF-α, in response to influenza-induced IFN-γ, drives lethal lung inflammation (16).

In the present study, we have performed single-cell RNA sequencing (scRNA-seq) to profile how IFN-γ signaling instructs lung inflammation. We demonstrate that IFN-γ induces a transcriptional shift in airway immune cells during IAV/MRSA coinfection. While IAV-induced *Ifng* expression is limited to the T cell cluster, myeloid cells are the main producers of *Tnfa* and *Il1b* in response to MRSA super-infection. These findings suggest an IFN-γ-mediated interplay between T cells and inflammatory cells in perpetuating immune dysfunction and inflammatory damage. Using a series of IFN-γ receptor 1 (*Ifngr1)* conditional knockout mice, we have further investigated the cellular pathways underlying IFN-γ-driven hypercytokinemia and their contributions to ARDS pathogenesis. We found that the proinflammatory cytokine response in the lung is primarily driven by IFN-γ signaling in myeloid cells, with the greatest contribution from mononuclear phagocytes. As such, mice with *Ifngr1* deletion in CD11c^+^ myeloid cells exhibited significantly attenuated lung inflammation and mortality after IAV/MRSA coinfection and gentamicin treatment.

## Materials and methods

### Murine model of viral and bacterial infection

Specific pathogen-free, C57BL/6 WT, *Ifng* ^-/-^, *Ifngr1^-/-^*, *Ifngr1* ^fl/fl^, *Lyz2* ^Cre^, *Cd11c* ^Cre^, *Cx3cr1* ^Cre^, *Mrp8* ^Cre^ and mTmG (17) mice were purchased from the Jackson Laboratory (Bar Harbor, ME) and bred at the University of Nebraska Medical Center (UNMC) and University of Texas Medical Branch (UTMB) following Animal Care and Use Committee (IACUC) guidelines. All animal experiments were approved by UNMC and UTMB, and all experiments were carried out in accordance with UNMC and UTMB Assurance of Compliance with PHS Policy on Humane Care and Use of Laboratory Animals, which is on file with the Office of Protection from Research Risks, NIH.

*Lyz2* ^Cre^, *Cd11c* ^Cre^, *Cx3cr1* ^Cre^, and *Mrp8* ^Cre^ reporter mice were generated by crossing mTmG mice with corresponding Cre^+^ mice. *Lyz2* ^Cre^_*Ifngr1* ^fl/fl^, *Cd11c* ^Cre^_*Ifngr1* ^fl/fl^, *Cx3cr1* ^Cre^_*Ifngr1* ^fl/fl^, and *Mrp8* ^Cre^_*Ifngr1* ^fl/fl^ mice were generated by crossing *Ifngr1* ^fl/fl^ mice with corresponding Cre^+^ mice.

Viral challenge was performed with a sublethal (0.2LD_50_) dose of PR8 administered i.n. to anesthetized, sex and age-matched adult mice in 50 µl of sterile PBS. Viral titers were determined by plaque assays on Madin-Darby canine kidney cell monolayers. Bacterial super-challenge was performed seven days later. To induce bacterial pneumonia, anesthetized mice were inoculated i.n. 4~6x10^8^ CFU/mouse of ATCC MRSA strain BAA-1695 in 50 μl of PBS. Bacterial burdens were measured by plating serial 10-fold dilutions of each sample onto blood agar plates.

### Bronchoalveolar lavage cell analysis

BAL fluid (BALF) samples were collected by making an incision in the trachea and lavaging the lung twice with 0.8 ml PBS, pH 7.4. Total leukocyte counts were determined using a hemocytometer.

For flow cytometric analysis, BALF cells were incubated with 2.4G2 mAb and stained with allophycocyanin-conjugated anti-CD11c (Biolegend), BUV395-conjugated or BV510-conjugated anti-CD11b (BD Biosciences), FITC-conjugated or PE-Cy7-conjugated anti-Ly6G (clone 1A8, Biolegend), PerCP-Cy5.5-conjugated (eBiosciences) or PE-conjugated anti-Ly6C (Biolegend), and BV421-conjugated or PE-conjugated anti-Siglec-F (Biolegend) mAbs. The stained cells were analyzed on a BD LSRII-green using BD FACSDiva and FlowJo software analysis.

### Determination of cytokine production by ELISA

BALF were harvested and assayed for IFN-γ, TNF-α, IL-1β, and IL-6 by ELISA using commercially available kits from BD Biosciences and R&D Systems (Minneapolis, MN).

### Single cell RNA sequencing

scRNA-seq was performed with the assistance of the UNMC Genomics Core. The core operates a 10x Genomics Chromium instrument for isolation of single cells and generation of Illumina compatible sequencing libraries (10x Genomics, Pleasanton, CA). The instrument captures individual cells using a gel bead in emulsion (GEM) technology. The droplets contain the reagents for reverse transcription and all transcripts within each cell are uniquely barcoded (all transcripts within a given cell contain the same barcode for cell identification) and Illumina compatible cDNA libraries created are for each cell within the given sample. Prior to beginning the capture, BALF cell suspensions were evaluated by light microscopy with respect to debris and viability. The single‐cell suspensions were diluted to the working concentration required to 8000 target cells captured, with *Ifngr1* ^-/-^ or *Ifng* ^-/-^ cells and corresponding WT controls each divided in half for library preparation. The pooled cells were captured, lysed, RNA reverse transcribed and barcoded using Chromium Single Cell 3’ Reagent Kits v3 reagents. Libraries are quantified by qPCR using the KAPA Library Quant Kit (Illumina) from KAPA Biosystems (Roche, Pleasonton, CA) and were loaded at a concentration of 1.3 pM on Illumina sequencing flowcells. The samples were sequenced on an Illumina NextSeq500 instrument (as recommended for 3’ end sequencing of transcripts from single cells).

### ScRNA-seq analyses

The 10x Genomics Cell ranger analysis pipeline (Version 3.0.0) was used for demultiplexing and generating the Feature-Barcode Matrices (18). Briefly, cellranger mkfastq was used to convert the raw base call (BCL) files of scRNA-seq into FASTQ files. Then cellranger count was used for alignment with reference genome mm10, filtering, and processing barcode data to create the feature-barcode matrices for each sample; cellranger aggr was used to normalize the samples to the same sequencing depth and then recomputing the feature-barcode matrices and perform analysis on the combined data including clustering and gene express analysis. We used 10x Genomics Loupe cell browser for differential expression analysis and creating *t*-SNE plots on which we mapped the marker gene expressions and the clustering results.

Additional bioinformatic analysis was performed using IPA (QIAGEN Inc.). We provided the DEG lists (fold change >4) to IPA for its core analysis and then overlaid with the global molecular network in the Ingenuity pathway knowledge base. As a result, the biological functions, disease annotations, and canonical pathways that were enriched in the DEG lists were identified and analyzed.

### Lung histology analysis

Mice were euthanized seven days after MRSA super-infection and the lungs were removed for histological analyses. Paraffin-embedded tissues were sectioned to a thickness of 5 µm and stained with hematoxylin and eosin (H&E) using standard methods. Whole mount H&E-stained lung tissues were scanned using Leica Aperio LV1 scanner and software, and semi quantitatively assessed at low-power (40×) for the proportion of parenchyma with alveoli containing intraluminal material (proteinaceous exudate and/or fibrin) in the background of interstitial expansion and inflammation. Each lung was scored by the relative amount of abnormal tissue as follows: normal-0, 1-25%-1, 26-50%-2, 51-75%-3, >76%-4 (15). Digital images were generated using Leica Biosystems Aperio ImageScope 12.

### Evaluation of airway damage

BALF were harvested and assayed for albumin by ELISA using a commercially available kit from Bethyl Laboratories (Montgomery, TX). Total protein levels and lactic acid dehydrogenase (LDH) activities in BALF were analyzed by a Micro bicinchoninic acid assay (BCA) protein assay kit (Thermo Scientific) and a LDH cytotoxicity assay kit (Thermo Scientific), respectively.

### Treatment with antibiotics

Mice were intraperitoneally (i.p.) injected with a therapeutic dose of vancomycin (300 mg/kg) or gentamicin (100 mg/kg) beginning 2 or 4 h after MRSA infection and then followed by 150 mg/kg/day for vancomycin or 50 mg/kg/day for gentamicin, respectively. All antibiotic treatment injections continued through day seven after MRSA infection. Minimum inhibitory concentration (MIC) values of antibiotics for MRSA BAA-1695 were ≤ 12.5 µg/ml for gentamicin and ≤ 1.17 µg/ml for vancomycin *in vitro* (14).

### Statistics

Significant differences between experimental groups were determined using a two-tailed Student *t*-test (to compare two samples), or an ANOVA analysis followed by Tukey’s multiple comparisons test (to compare multiple samples) in GraphPad Prism 9 (La Jolla, CA). Survival analyses were performed using the log-rank test. For all analyses, a *P* value <0.05 was considered to be significant.

## Results

### IFN-γ receptor signaling promotes hypercytokinemia during post-influenza MRSA pneumonia

We have developed a mouse model of ALI induced by post-influenza MRSA infection (15). This secondary bacterial pneumonia model replicates the poor outcome of patients with pneumonic ARDS, despite appropriate antibiotic treatment (8, 13, 19–21). Specifically, C57BL/6 mice were challenged with IAV strain A/PR8/1934 followed by intranasal (i.n.) super-infection of MRSA seven days later (15). IAV/MRSA coinfected mice were treated daily with antibiotic vancomycin or gentamicin for effective bacterial control. However, antibiotic therapy alone is not sufficient to improve animal survival (14, 16).

**Figure 1:**
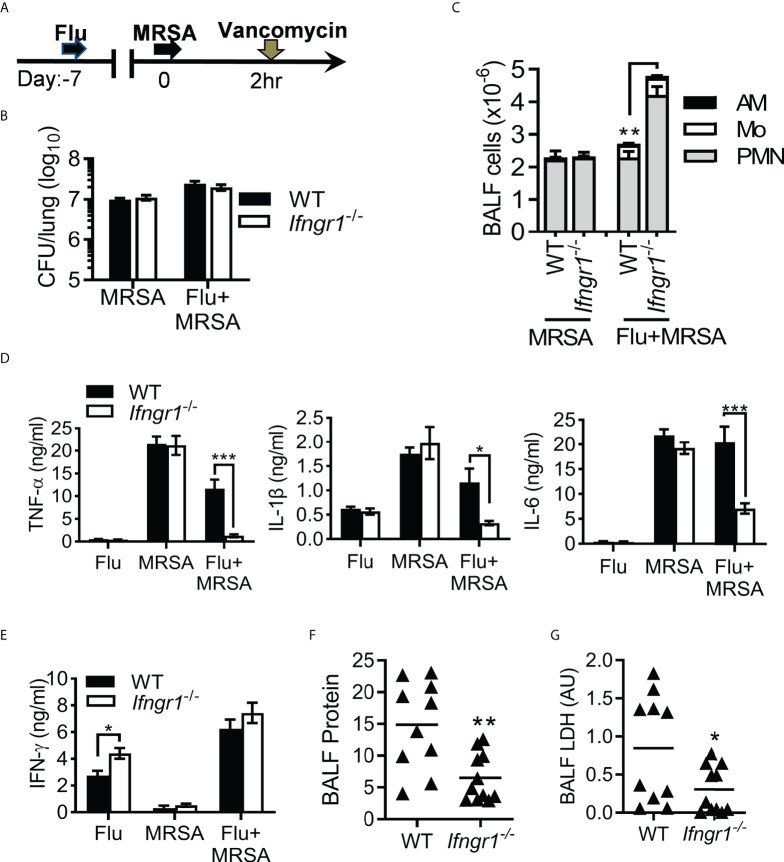
IFN-γ signaling induces hypercytokinemia and acute lung damage. **(A)** Experimental scheme. Mice were infected with PR8 virus and then challenged with MRSA on day seven post-influenza infection. All PR8-infected (Flu), MRSA-infected (MRSA), or coinfected mice (Flu+MRSA) were treated with vancomycin 2 h after MRSA or PBS inoculation. **(B)** Lung bacterial burdens, **(C)** BALF levels of (mean±SD, n≥4) AM, monocytes (Mo) and neutrophils (PMN), **(D)** TNF-α, IL-1β and IL-6, and **(E)** IFN-γ at 24 h after PR8 and/or MRSA infection. **(F)** BALF levels of protein and **(G)** lactate dehydrogenase (LDH) at 7 dps in WT and *Ifngr1^-/-^* mice (mean±SEM, n≥5) after PR8/MRSA coinfection. Data in **(F)** represented protein levels relative to naïve mice. AU, arbitrary unit. *P<0.05, **P<0.01, ***P<0.001, *t*-test. Data shown are representative of at least two independent experiments.

We have recently shown in this model that combining antibiotic treatment with IFN-γ neutralizing antibodies prevents hypercytokinemia and thereby improves protection against ALI and animal mortality (16). This IFN-γ-driven excessive lung inflammation was further verified in the global *Ifngr1* gene-deficient (*Ifngr1* ^−/−^) mouse model (**Figure 1A**). Vancomycin-treated WT and *Ifngr1* ^-/-^ mice exhibited similar bacterial burdens after MRSA infection alone and IAV/MRSA coinfection (**Figure 1B**). Interestingly, compared with respective WT controls, *Ifngr1* ^-/-^ mice exhibited increased airway neutrophil accumulation after coinfection (**Figure 1C**). In contrast, acute proinflammatory cytokines TNF-α, IL-1β and IL-6 were diminished in coinfected *Ifngr1* ^-/-^ mice (**Figure 1D**), while their IFN-γ production was not significantly different from coinfected WT controls (**Figure 1E**). Importantly, acute lung damage was attenuated in *Ifngr1* ^-/-^ mice, as indicated by significantly reduced BALF protein and LDH levels 7 days post-MRSA super-infection (dps) (**Figures 1F-G**). Together, these results establish that IFN-γ receptor signaling promotes hypercytokinemia and lung damage during secondary MRSA pneumonia.

### IFN-γ signaling transforms the transcriptomic landscape of airway immune cells

**Figure 2:**
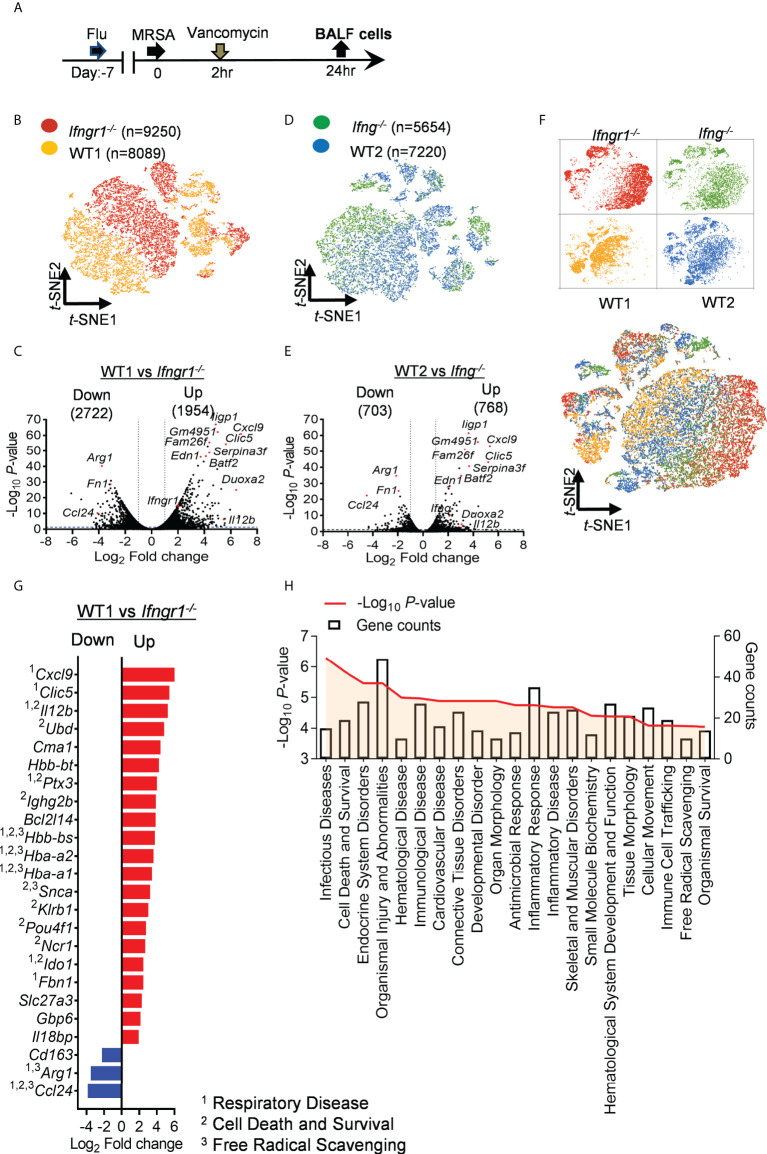
IFN-γ induces fundamental changes in the airway transcriptomic landscape. **(A)** Experimental scheme for scRNA-seq analysis. **(B)** *t*-SNE projections distinguishing *Ifngr1* ^-/-^ cells (red) from WT controls (brown) in the same experiment. Each dot represents a cell. **(C)** Volcano plot for DEGs in WT cells as compared to *Ifngr1* ^-/-^ datasets. **(D)** *t*-SNE projections and **(E)** volcano plot for DEGs in WT cells (blue) as compared to *Ifng* ^-/-^ datasets (green). **(F)** *t*-SNE projections showing overlap among *Ifngr1* ^-/-^, *Ifng* ^-/-^ and WT cells. Four datasets were combined from two independent experiments. **(G)** Diagram showing top DEG (fold change >4) associated with inflammatory disease conditions, overlapping with genes related to ^1^respiratory disease, ^2^cell death and survival, and ^3^free radical scavenging in WT mice as compared to corresponding *Ifngr1* ^-/-^ animals. Red: upregulated; blue: downregulated in WT cells. **(H)** Representative classification of diseases and functions mediated by IFN-γ receptor signaling. Categories are shown in terms of the –log_10_ (*P*-value), as represented by the left y-axis, and the number of DEGs counted, represented by the right y-axis.

To determine the impact of IFN-γ signaling on the lung inflammatory profile, we performed scRNA-seq of bronchoalveolar lavage fluid (BALF) cells 24 h after IAV/MRSA coinfection of *Ifngr1* ^-/-^ and WT mice side-by-side (**Figure 2A**). The clustering analysis revealed a clear segregation of *Ifngr1* ^-/-^ cell populations from WT controls (**Figure 2B**). We next compared gene expressions between WT and *Ifngr1* ^-/-^ cells. A total of 4676 differentially expressed genes (DEGs, *P*<0.05), including 1954 upregulated and 2722 downregulated, were identified in WT cells (**Figure 2C**).

To determine whether IFN-γ signaling is solely responsible for the fundamental changes in the transcriptomic landscape, we further performed scRNA-seq of BALF cells from *Ifng* ^-/-^ mice as compared with corresponding WT controls (**Figures 2D-E**). Like *Ifngr1* ^-/-^ mice, there was very limited overlap between *Ifng* ^-/-^ and corresponding WT landscapes on the *t*-SNE plot. Importantly, the combined analysis of four datasets revealed that *Ifngr1* ^-/-^ and *Ifng* ^-/-^ cell clusters from two independent experiments were more intermixed than with their respective WT controls (**Figure 2F**). These results establish that IFN-γ signaling induces fundamental changes in the airway transcriptome after IAV/MRSA coinfection.

Using the ingenuity pathways analysis (IPA) system with a threshold of −log (*P*−value) > 4 and DEG counts ≥ 10, we found that IFN-γ-activated molecular and cellular functions were associated with cell death and survival, and inflammatory diseases (**Figure 2G**). Further disease and function analysis indicates that apart from inflammatory response, IFN-γ receptor signaling is also involved in multiple organ/tissue disorders, organismal injury and abnormalities, as well as organismal survival (**Figure 2H**). These fundamental changes in the transcriptomic profile suggest that IFN-γ plays a central role in causing immune cell dysfunction and inflammatory tissue damage during post-influenza MRSA pneumonia.

### IFN-γ upregulates monocyte and macrophage expression of genes associated with inflammatory diseases

**Figure 3:**
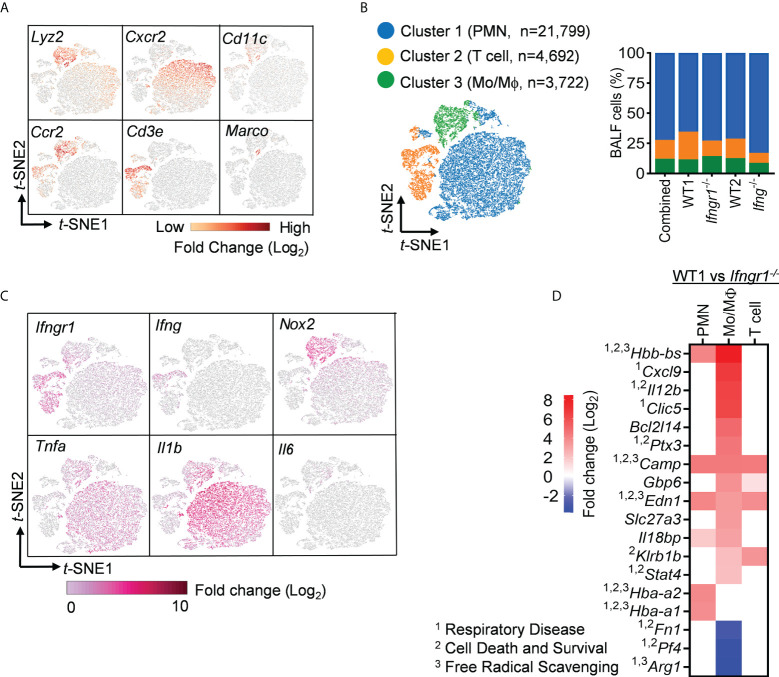
IFN-γ induces robust upregulation of inflammatory disease genes in macrophages and monocytes. **(A)** Visualization of expression of cell type signature genes and **(B)** *t*-SNE plot and the percentages of three major clusters in scRNA-seq datasets combined of WT, *Ifngr1* ^-/-^ and *Ifng* ^-/-^ BALF cells. **(C)** Single-cell expression of six selected inflammatory genes. **(D)** Diagram showing top DEGs (fold change > 4) associated with inflammatory disease conditions in WT cell clusters as compared with corresponding *Ifngr1* ^-/-^ cells, overlapping with genes related to ^1^respiratory disease, ^2^cell death and survival, and ^3^free radical scavenging. Red: upregulated; blue: downregulated in WT cells.

To further differentiate the effect of IFN-γ signaling on airway immune cell subsets, we performed pairwise comparison of *Ifngr1* ^-/-^ and *Ifng* ^-/-^ transcription profiles with corresponding WT controls within three major cell clusters, representing neutrophils (*Lyz2* ^+^ *Cxcr2* ^+^), T cells (*Cd3* ^+^), and monocytes/macrophages (Mo/Mψ, *Lyz2* ^+^ *Ccr2* ^+^) (**Figures 3A-B**). Of note, *Cd11c* ^+^ *Marco* ^+^ alveolar macrophages (AM) contributed to the Mo/Mψ cluster but only a minor proportion (**Figure 3A**).

In line with IFN-γ-induced fundamental changes in the airway transcriptome, we detected broad *Ifngr1* expression in these immune cells (**Figure 3C**). As expected, *Ifng* expression was limited to the T cell cluster. On the other hand, inflammatory cells, as indicated by their *Lyz2* and *Nox2* expression, appeared to be the predominant producers of *Tnfa* and *Il1b*. Surprisingly, *Il6* mRNA was detected only in a small number of cells in the Mo/Mψ cluster. Thus, it is possible that IL-6 is primarily produced by lung stromal cells rather than immune cells. Together, these findings imply an IFN-γ-initiated interplay of T cells, inflammatory cells, and presumably, lung tissue cells in driving hypercytokinemia after IAV/MRSA coinfection.

**Figure 4:**
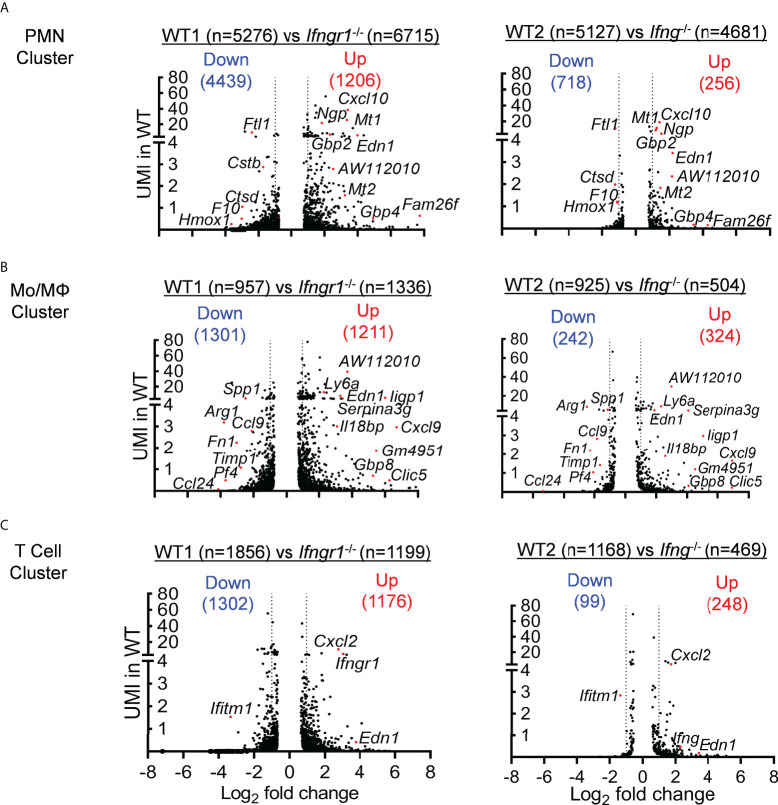
IFN-γ primarily induces transcriptomic changes in myeloid cells. ScRNA-seq analysis of DEGs (*P*<0.05) in WT **(A)** neutrophil, **(B)** Mo/Mψ, and **(C)** T cell clusters as compared with corresponding *Ifngr1* ^-/-^ (left) and *Ifng* ^-/-^ (right) subset in two independent experiments. Volcano plots show the logarithm of fold change (log2) on x-axis and UMI of each transcript on y-axis.

A further IPA analysis of these IFN-γ-induced DEGs revealed an enrichment of top genes, *i.e.*, Log (*P*−value) > 4 and unique molecular identifier (UMI) > 0.1, associated with inflammatory response and organismal injury, overlapping with genes related to respiratory disease, cell death and survival, and free radical scavenging (**Figure 3D**). It is noteworthy that these top DEGs associated with respiratory inflammation, *e.g*. > 50-fold increased *Cxcl9* and *Il12b* and >15-fold decreased *Arg1* in WT cells, appeared to be most active within myeloid cells, particularly the Mo/Mψ cluster (**Figure 4** **)**. This suggests that in response to IAV/MRSA coinfection, T cell-derived IFN-γ may directly activate myeloid cells to enhance their proinflammatory properties.

### IFN-γ signaling in myeloid cells drives hypercytokinemia and lethal IAV/MRSA coinfection

**Figure 5:**
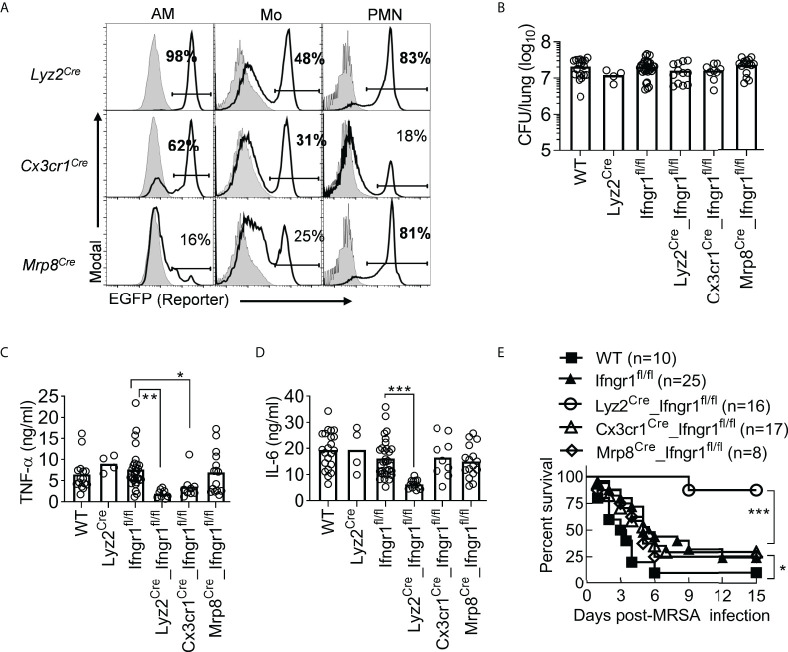
IFN-γ signaling in myeloid cells drives TNF-α and IL-6 hyperproduction and animal mortality. **(A)** Flow cytometry analysis of percentages (mean of ‗3 mice/group) of EGFP-expressing AM, monocytes (Mo), neutrophils (PMN) in *Lyz2* ^Cre^_mTmG, *Cx3cr1* ^Cre^_mTmG, and *Mrp8* ^Cre^_mTmG mice (black line), and mTmG controls (solid grey) 24 h after PR8/MRSA coinfection. **(B)** Lung bacterial burdens, **(C)** BALF TNF-α and **(D)** IL-6 levels at 24 h, and **(E)** animal survival of WT, *Lyz2* ^Cre^, *Ifngr1* ^fl/fl^, *Lyz2* ^Cre^_*Ifngr1* ^fl/fl^, *Cx3cr1* ^Cre^_*Ifngr1* ^fl/fl^, and *Mrp8* ^Cre^_*Ifngr1* ^fl/fl^ mice after PR8/MRSA coinfection. All mice were treated with vancomycin starting 2 h after MRSA infection. *P<0.05, **P<0.01, ***P<0.001, Tukey’s multiple comparisons test **(B-D)** or log-rank test **(E)**. Data shown were combined from more than two independent experiments.

To determine the contribution of different myeloid cell subsets to coinfection pathogenesis, we developed cre-lox mouse models with *Ifngr1* deletion (22) in myeloid cells (*Lyz2* ^Cre^) (23), Mo/Mψ (*Cx3cr1* ^Cre^) (24), and neutrophils (*Mrp8* ^Cre^) (25) (**Figure 5A**). These conditional *Ifngr1* knockout mice displayed similar lung bacterial burdens as WT controls after IAV/MRSA coinfection and vancomycin treatment (**Figure 5B**). Nonetheless, deletion of *Ifngr1* in myeloid cells (*Lyz2^Cre^* _*Ifngr1* ^fl/fl^) led to nearly 6- and 10-fold decreased TNF-α and IL-6 production, respectively (**Figures 5C-D**). These results indicate that IFN-γ-activated myeloid cells are responsible for driving hypercytokinemia after coinfection. Mice carrying *Ifngr1* deficiency in Mo/Mψ (*Cx3cr1* ^Cre^_*Ifngr1* ^fl/fl^) also exhibited reduced TNF-α production; their IL-6 production, however, was comparable to *Ifngr1* ^fl/fl^ controls. In contrast, deletion of *Ifngr1* primarily in neutrophils (*Mrp8* ^Cre^_*Ifngr1* ^fl/fl^) had no significant effect on TNF-α or IL-6 response. These results suggest that IFN-γ-activated mononuclear phagocytes play a more key role in TNF-α hyperproduction.

In agreement with their diminished proinflammatory cytokine response, coinfected *Lyz2^Cre^* _*Ifngr1* ^fl/fl^ mice exhibited significantly improved survival with vancomycin treatment, as compared with mice carrying *Ifngr1* deletion limited to either Mo/Mψ (*Cx3cr1* ^Cre^_*Ifngr1* ^fl/fl^) or neutrophils (*Mrp8* ^Cre^_*Ifngr1* ^fl/fl^) (**Figure 5E**). These results suggest that IFN-γ-activated myeloid cells are responsible for driving hypercytokinemia and lethal IAV/MRSA coinfection.

### IFN-γ directly activates mononuclear phagocytes to promote TNF-α hyperproduction and inflammatory lung damage

**Figure 6:**
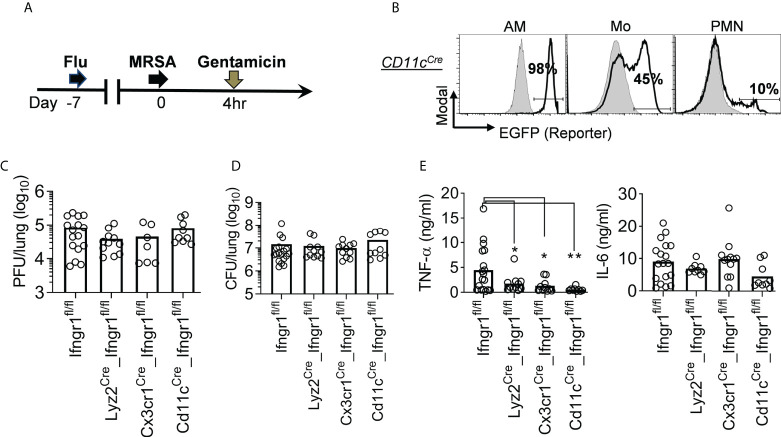
IFN-γ directly activates mononuclear phagocytes to promote TNF-α production. **(A)** Experimental scheme. All mice were treated with gentamicin starting 4 h after MRSA super-infection. **(B)** Flow cytometry analysis of percentages (mean of ‗3 mice/group) of EGFP-expressing AMs, monocytes (Mo), and neutrophils (PMN) in *Cd11c* ^Cre^_mTmG reporter mice (black line), and mTmG controls (solid grey) 24 h after PR8/MRSA coinfection. **(C-D)** Lung viral and bacterial burdens and **(E)** TNF-α and IL-6 levels 24 h after PR8/MRSA coinfection of *Ifngr1* ^fl/fl^, *Lyz2* ^Cre^_*Ifngr1* ^fl/fl^, *Cx3cr1* ^Cre^_*Ifngr1* ^fl/fl^, and *Cd11c* ^Cre^_*Ifngr1* ^fl/fl^ mice. *P<0.05, **P<0.01, Tukey’s multiple comparisons test. Data shown are representative of two independent experiments.

Our results indicate that IFN-γ-activated mononuclear phagocytes play a dominant role in driving TNF-α hyperproduction (**Figure 5C**). Considering that CX3CR1 is also expressed in lymphocytes such as NK and T cells (24), we further developed *Cd11c^Cre^* _*Ifngr1* ^fl/fl^ mouse model (26) to induce *Ifngr1* deletion specifically in CD11c^+^ mononuclear phagocytes (**Figure 6**). We have previously shown that compared with bacteriolytic vancomycin, treatments with protein synthesis inhibitor antibiotics linezolid and gentamicin provide better protection against *S. aureus* α-toxin-induced direct lung injury during IAV/MRSA coinfection (14, 16). Thus, we utilized this more sensitive lung inflammatory damage model, *i.e.*, IAV/MRSA coinfection plus gentamicin treatment, to further differentiate the role of myeloid cell subsets in lung injury (**Figure 6A**).

**Figure 7:**
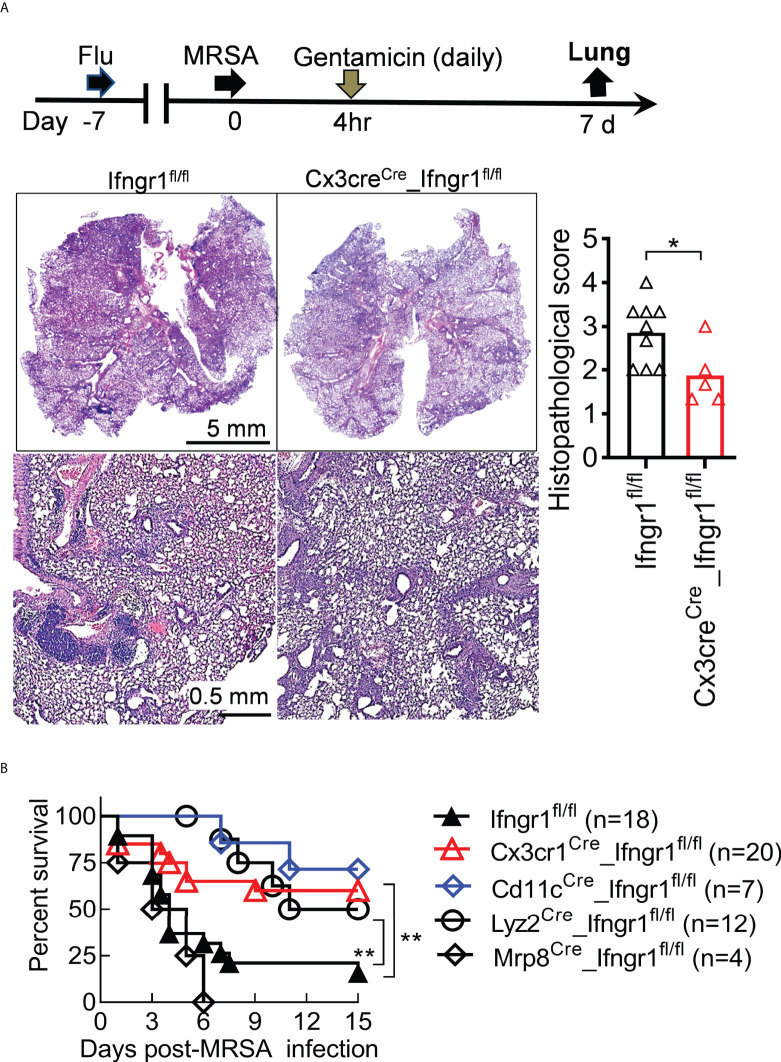
IFN-γ directly activates mononuclear phagocytes to promote acute lung damage. **(A)** Experimental scheme, and lung histopathology (H&E) and histopathologic scores (each symbol represents one mouse) at 7 dps. **(B)** Survival of *Ifngr1* ^fl/fl^, *Cx3cr1* ^Cre^_*Ifngr1* ^fl/fl^, *Cd11c* ^Cre^_*Ifngr1* ^fl/fl^, *Lyz2* ^Cre^_*Ifngr1* ^fl/fl^ and *Mrp8* ^Cre^_*Ifngr1* ^fl/fl^ mice after PR8/MRSA coinfection. *P<0.05, **P<0.01, *t*-test **(A)**, or log-rank test **(B)**. Data shown are representative of at least two independent experiments.

Deletion of *Ifngr1* in CD11c^+^ myeloid cells had no significant impact on lung viral and bacterial control (**Figures 6C-D**). Conversely, BALF level of TNF-α was significantly reduced in *Cd11c^Cre^* _*Ifngr1* ^fl/fl^ animals, in a pattern similar to *Lyz2^Cre^* _*Ifngr1* ^fl/fl^ and *Cx3cr1* ^Cre^_*Ifngr1* ^fl/fl^ mice (**Figure 6E**). This finding suggests that IFN-γ signaling in monocytes and macrophages drives TNF-α production. In line with that, *Cx3cr1* ^Cre^_*Ifngr1* ^fl/fl^ mice exhibited reduced histopathological scores at 7 dps, suggesting that IFN-γ signaling in mononuclear phagocytes are responsible for triggering severe lung damage in *Ifngr1* ^fl/fl^ controls (**Figure 7A**). Importantly, gentamicin treatment significantly improved the survival of *Cd11c^Cre^* _*Ifngr1* ^fl/fl^ and *Cx3cr1* ^Cre^_*Ifngr1* ^fl/fl^ mice after IAV/MRSA coinfection (**Figure 7B**). These findings indicate that IFN-γ directly activates monocytes and macrophages to elicit TNF-α production and lethal lung inflammation during post-influenza MRSA pneumonia.

## Discussion

In the present study, we have investigated the signaling pathway underlying IFN-γ-driven lethal lung inflammation during IAV/MRSA coinfection. IAV infection is known to induce prominent IFN-γ production by T cells. Here we show that IFN-γ signaling induces fundamental changes in the airway transcriptome in response to MRSA infection, characterized by upregulations of genes associated with immune cell dysfunction and inflammatory tissue damage. Using a series of *Ifngr1* conditional knockout mouse models, we further demonstrate that IFN-γ signaling in myeloid cells, particularly mononuclear phagocytes, directly activates TNF-α production and lethal lung damage. Collectively, these findings, ranging from cell-intrinsic gene regulation to overall impact on disease outcome, suggest that IFN-γ induces hyperresponsiveness of monocytes and macrophages to trigger ARDS pathogenesis during influenza-complicated MRSA infection.

Secondary bacterial infection following influenza is known to cause severe pneumonia. However, the reported studies generally focus on how IAV infection impairs antibacterial immunity and thereby increases host susceptibility to secondary bacterial infection (27–34). In the current model, IAV/MRSA-coinfected mice were treated with adequate antibiotics to mediate bacterial killing. Thus, our model mimics the clinical scenario where critically ill patients develop ARDS and even death, despite appropriate antimicrobial therapies. This unique model allows us to investigate how dysregulated inflammatory response contributes to ARDS pathogenesis, independent of defective bacterial control.

Using *Ifngr1* conditional knockout mouse models, we show that IFN-γ signaling in myeloid cells is sufficient to trigger hypercytokinemia and ARDS pathogenesis. In agreement, our scRNA-seq data demonstrate that IFN-γ-induced gene upregulation in WT myeloid cells presents an inflammatory signature, *e.g*., *Cxcl9* in monocytes and *Cxcl10 in* neutrophils. Of particular interest, CXCL10-CXCR3 signaling has been shown to promote oxidative burst in neutrophils, thereby leading to fulminant lung inflammation (35–37). In contrast, genes upregulated in *Ifngr1* ^-/-^ and *Ifng* ^-/-^ cells, such as *Arg1* and *Cd163* genes, are associated with immunoregulatory function and tissue repair (38).

The hallmark of ARDS is inflammatory cell accumulation in the lung. Influenza triggers intensive recruitment of inflammatory monocytes (39), while MRSA infection induces heightened neutrophil infiltration. Interestingly, IAV/MRSA coinfection-induced neutrophilic infiltration increases in the global *Ifngr1* knockout mice (**Figure 1B**). Nonetheless, deletion of *Ifngr1* only in myeloid cells did not appear to have a significant impact on the airway neutrophil accumulation (data not shown). These observations suggest that rather than promoting inflammatory infiltration, dysregulation of inflammatory cell function is the key mechanism in IFN-γ-driven alveolar inflammation and animal mortality.

Mononuclear phagocytes, particularly macrophages and monocytes, are known for their functional plasticity in response to cytokine milieu (40). In agreement, here we show that mononuclear phagocytes play a dominant role in driving TNF-α production and ARDS pathogenesis. Nonetheless, future studies are necessary to establish whether IFN-γ signaling promotes TNF-α production directly by this inflammatory cell subset or indirectly by epithelial cells. In contrast, deletion of *Ifngr1* only in neutrophils (*Mrp8* ^Cre^_*Ifngr1* ^fl/fl^) has no significant effect on inflammatory cytokine response and animal mortality (**Figure 6**). These results suggest that the regulatory effect of IFN-γ on neutrophils is largely indirect, again highlighting the significance of IFN-γ-activated mononuclear phagocyte subset in ARDS pathogenesis.

Compared to the magnitude of inflammatory cell influx, necrotic cell death is more important in causing acute lung injury. Our previous study has demonstrated that oxidative stress derived from phagocyte NADPH activity promotes necrotic cell death (15). Inflammatory cytokines are responsible for activation of inflammatory cell recruitment and oxidative burst. Interestingly, MRSA infection alone induces intensive production of proinflammatory cytokines, independent of IFN-γ signaling (**Figure 1D-E**). In this case, however, antibiotic therapy is effective in improving animal survival (14). It has been reported that IFN-γ, in synergy with TNF-α, triggers inflammatory cell death and directly contributes to lethal tissue damage in SARS-CoV-2 infection and cytokine storm syndromes. Furthermore, blocking both IFN-γ and TNF-α with specific antibodies protected against inflammatory cell death and animal mortality from cytokine shock, sepsis, and hemophagocytic lymphohistiocytosis, in addition to SARS-CoV-2 infection (41). Thus, in addition to stimulating TNF-α production, IFN-γ is likely involved in other immunopathogenic aspects of IAV/MRSA coinfection, such as promoting inflammatory cell death.

In summary, our findings from the current study, ranging from cell-specific gene regulation to overall disease outcome, indicate that IFN-γ directly activates mononuclear phagocytes to trigger TNF-α hyperproduction and ARDS pathogenesis during IAV/MRSA coinfection. Considering the prominent IFN-γ-associated cytokine storm in various infectious and autoinflammatory conditions, strategies to target these early events represent potential therapeutic intervention for attenuating inflammatory lung damage in ARDS-prone diseases.

## Data availability statement

The data presented in the study are deposited in the GEO repository, accession number GSE212402.

## Ethics statement

The animal study was reviewed and approved by University of Nebraska Medical Center (UNMC) and University of Texas Medical Branch (UTMB) Animal Care and Use Committee (IACUC).

## Author contributions

Conceived and designed the experiments: KS. Performed the experiments: AV, MM, MU, SP, CB and SS. Analyzed the data: KS and MN. Wrote the paper: KS and MM. All authors contributed to the article and approved the submitted version.

## Funding

This work was funded by NIH grant R01 HL118408 to KS. We thank Dr. James Eudy for advice on single-cell sequencing. The UNMC DNA Sequencing Core receives partial support from P20GM103427, P30GM110768, and P30CA036727. We also thank the Bioinformatics and Systems Biology Core at UNMC for providing “NAME” data analysis services, which receives support from Nebraska Research Initiative (NRI) and NIH (2P20GM103427 and 5P30CA036727). The authors also thank UTMB Anatomic Pathology.

## Conflict of interest

The authors declare that the research was conducted in the absence of any commercial or financial relationships that could be construed as a potential conflict of interest.

## Publisher’s note

All claims expressed in this article are solely those of the authors and do not necessarily represent those of their affiliated organizations, or those of the publisher, the editors and the reviewers. Any product that may be evaluated in this article, or claim that may be made by its manufacturer, is not guaranteed or endorsed by the publisher.
